# An evaluation of the coax monopole antenna as a transmit array element for head imaging at 14 T

**DOI:** 10.1002/mrm.30464

**Published:** 2025-02-18

**Authors:** Lyanne M. I. Budé, Koen Vat, Ingmar J. Voogt, Irena Zivkovic, Alexander J. E. Raaijmakers

**Affiliations:** ^1^ Department of Electrical Engineering Eindhoven University of Technology Eindhoven The Netherlands; ^2^ Department of Biomedical Engineering Eindhoven University of Technology Eindhoven The Netherlands; ^3^ Wavetronica B.V. Utrecht The Netherlands; ^4^ Division of Imaging and Oncology UMC Utrecht Utrecht The Netherlands

**Keywords:** engineering, RF coil arrays, ultrahigh field MRI

## Abstract

**Purpose:**

In comparison to dipole antennas, the coax monopole antenna (CMA) diminishes the possibility of cable‐coil coupling. This greatly facilitates cable routing in spatially restricted environments, such as head coil arrays. With the outlook of a 14T MRI system being installed at the Donders Center in Nijmegen, the Netherlands, this study aims to optimize the CMA for an eight‐channel head array at 14 T and compare its performance with an array of fractionated dipole antennas.

**Methods:**

Both antenna designs were optimized for head imaging at 14 T using single‐channel finite‐difference time‐domain (FDTD) simulations at 596 MHz. Eight‐channel simulations were then used on a human model to evaluate $B_{1}^{+}$ and specific absorption rate (SAR) distributions. For both antenna types, prototype arrays were built by placing eight elements on a 26‐cm‐diameter cylindrical holder. These prototype arrays were used for S_11_ and S_12_ evaluation.

**Results:**

The optimal dimensions of the CMA were a length of 20 cm and a gap position of 4 cm. The fractionated dipole was optimal for a length of 25 cm. Evaluation of 100 000 random shims revealed that the CMA performs with lower SAR efficiency, although the SAR efficiencies are similar in CP mode. Measured S_11_ and S_12_ levels were both lower for the CMA.

**Conclusion:**

The coax monopole would be an excellent candidate for head coil arrays at 14T MRI. Although the CMA is expected to perform with lower SAR efficiency than the fractionated dipole, its single‐ended design will facilitate elements placement and cable‐routing, especially in a spatially restricted environment.

## 1 INTRODUCTION

In the MR community, increasing the B_0_ field has always been a major aim due to the expected increase in signal‐to‐noise ratio and contrast‐to‐noise ratio.^1^ Although many 7T MR systems exist, operating systems beyond 7 T are limited. To take this even further, an open consortium has been formed in the Netherlands, consisting of several academic parties to form the Dutch national 14T MRI initiative in medical sciences (DYNAMIC in short).^2^

DYNAMIC aims to develop a body‐wide 14T MR system for installation at the Donders Institute for Brain, Cognition, and Behavior in Nijmegen. Compared with the ultrahigh‐field standard of 7 T, a factor 3 increase in sensitivity is anticipated.^2^ For brain imaging, an 8‐channel and a 16‐channel transmit array will be developed, which will be combined with dedicated receive arrays. This research focuses on the 8‐channel transmitter.

At ultrahigh field strengths, the wavelength in tissue of the $B_{1}^{+}$ (transmit) field becomes very short (e.g., 6 cm at 14 T). These short wavelengths cause strong radiofrequency attenuation and interference patterns, resulting in so‐called dark spots, where no signal is generated, and potentially increased specific absorption rate (SAR) levels.^3^ Multichannel transmit arrays are therefore used to steer the $B_{1}^{+}$ to the region of interest. In past years, many groups have worked on optimizing transmit arrays for imaging at ultrahigh field strengths. Particularly dipole antennas have been shown to have attractive features.^4^, ^5^, ^6^, ^7^ For head imaging at 14 T, the fractionated dipole has been considered a potential candidate due to its high SAR efficiency, high field homogeneity, and robustness toward loading variations,^5^, ^8^

Despite the advantageous features, dipole antennas in general have the disadvantage that they are fed in the center. This means that for arrays with tight spatial constraints, such as head arrays, the feeding cable must be placed parallel to one of the conductive legs of the antenna. The strong longitudinal E‐fields that are present close to the antenna can then induce currents on the shield of the feeding cable, which causes cable‐coil coupling.^9^ To avoid such coupling, the feeding cable must be placed carefully, making cable routing difficult.

Recently, the coax monopole antenna (CMA) has been introduced as a transmit element for ultrahigh field MRI.^10^ The CMA consists of a coaxial cable connected directly to the feeding cable, with an interruption in the coaxial shield, which makes the cable radiative. An inductor at the end matches the antenna to 50 Ω, eliminating the need for a matching network. A cable trap enforces the desired antenna length. In comparison to center‐fed dipole antennas, the single‐ended design of this antenna strongly alleviates the possibility of cable‐coil coupling.^9^ Its SAR efficiency, defined as the $B_{1}^{+}$ level divided by the square root of the peak local SAR, averaged over 10 g of tissue, was demonstrated to be higher or equivalent to other proposed antennas. Multitransmit head arrays are commonly combined with multichannel receive arrays, which make the design quite spatially restrictive. The CMA would therefore be an attractive candidate for a multitransmit head‐coil array design at 14 T. This research focuses on a comparison between the CMA and the fractionated dipole antenna for an eight‐channel transmit array for head imaging at 14 T.

The antenna design was optimized for imaging at 596 MHz using single‐channel electromagnetic simulations. Subsequently, single‐channel simulations were used to evaluate the performance of the optimized CMA in comparison to a fractionated dipole antenna, which was similarly optimized for this frequency. In addition, eight‐channel head coil array simulations were performed on a human model for both antenna designs. Finally, two head coil arrays consisting of eight elements of each antenna type were manufactured. For these arrays, reflection and coupling levels were evaluated with eight‐channel S‐parameter measurements.

## 2 METHODS

### 2.1 Single‐channel simulations

The performances of the CMA and fractionated dipole antenna were compared with each other in terms of $B_{1}^{+}$ efficiency ($B_{1}^{+}$ normalized to 1 W of conducted power), SAR levels, and SAR efficiency ($B_{1}^{+}/\sqrt{\mathrm{SA}R_{10g,\max}}$). To do so, both antenna designs needed to be optimized for head imaging at 14 T.

**FIGURE 1:**
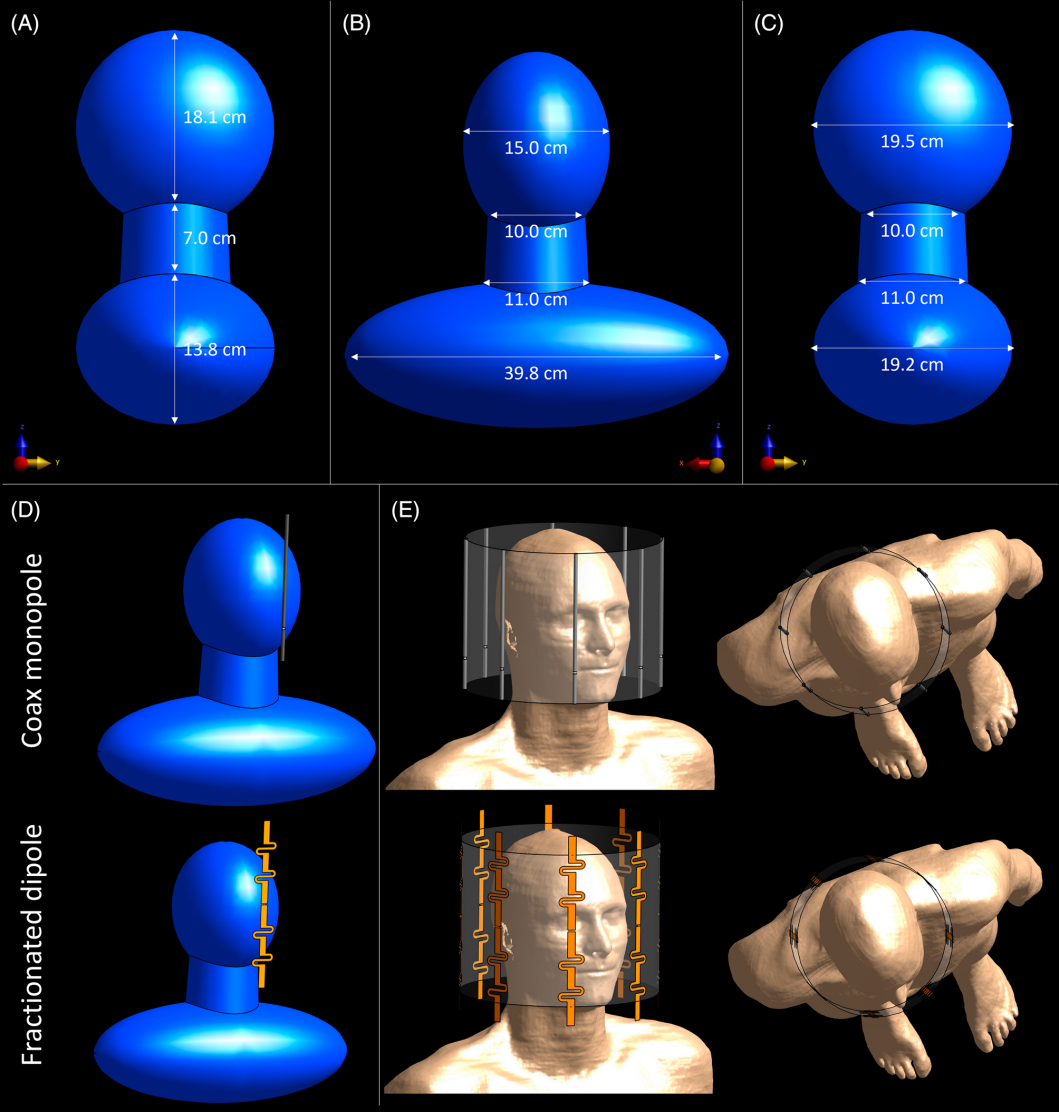
Homogeneous head‐shaped phantom modeled after the dimensions of Duke. (A) Dimensions in feet–head (FH) direction. (B) Dimensions in left–right (LR) direction. (C) Dimensions in the anterior–posterior (AP) direction. The head is modeled with an ellipsoid with FH, LR, and AP dimensions of 18.1, 15.0, and 19.5 cm, respectively. The neck consists of a cone with an upper diameter of 10.0 cm, lower diameter of 11.0 cm, and a height of 7 cm. The shoulders are modeled with an ellipsoid with FH, LR, and AP dimensions of 13.8, 39.8, and 19.2 cm, respectively. (D) Simulation geometry for single‐channel simulations for the coax monopole and fractionated dipole. (E) Simulation geometry for eight‐channel head simulations on human model Duke using coax monopole and fractionated dipole antennas. The antennas were placed in a circular array with a diameter of 26 cm.

For the optimization, finite‐difference time‐domain simulations were performed in *Sim4Life* (Zurich Medtech, Switzerland). Within this environment, a homogeneous head phantom was created with geometrical dimensions modeled after the head dimensions of the human model Duke from the virtual family.^11^ It consists of two ellipsoids and a cone to closely mimic the extent of the head, neck, and shoulders of Duke. Its anterior–posterior, left–right, and feet–head dimensions are 19.6, 39.8, and 38.9 cm, respectively. A more detailed representation of the model is depicted in Figure 1. Phantom properties were chosen to mimic the average brain at 14 T (σ = 0.66 S/m, ε_r_ = 47.52^12^). The single antenna to be evaluated was placed 50 mm in front of the phantom, measured from the antenna center to the surface of the phantom. For all single‐channel simulations, the phantom was voxelized at a maximum cell size of 5 × 5 × 5 mm^3^, and a convergence criterion of −50 dB was used.

#### 2.1.1 CMA

**FIGURE 2:**
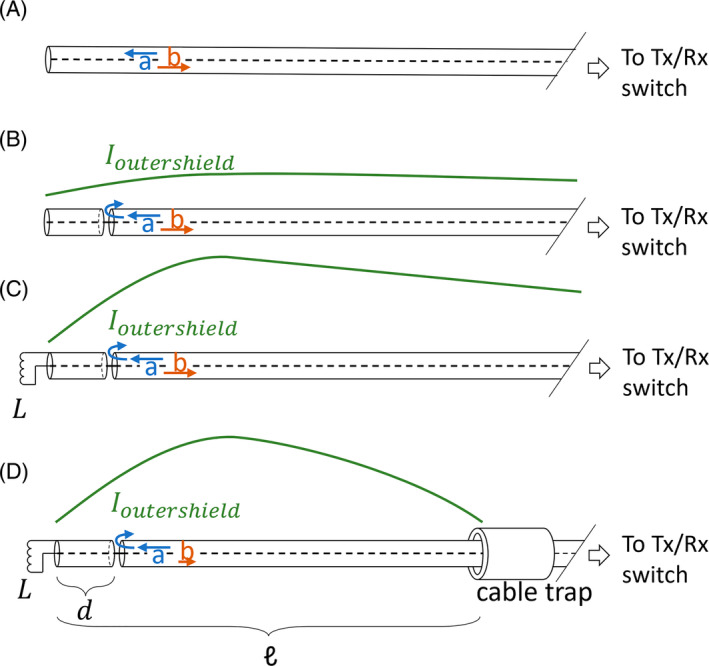
(A) Driving an open‐ended coax cable antenna results in full reflection and absence of a current on the outside of the shield (i.e., no $B_{1}^{+}$ field is generated). (B) An interruption in the shield at gap position *d* allows current to flow to the outside of the shield. (C) An inductor *L* at the end of the cable reduces the reflections and matches the antenna to 50 Ω. (D) A cable trap forces the outside current to zero at the desired antenna length ℓ. Rx, receive; Tx, transmit.

The CMA, as presented schematically in Figure 2, features an interruption in the shield of the coaxial cable at gap position *d*, to allow the current to flow to the outside of the shield. An inductor *L* at the antenna's end provides matching to 50 Ω. A cable trap is used to enforce the desired antenna length ℓ. As shown in a previous study, changing the parameters *L*, *d*, and ℓ determines the antenna's efficiency and matches the antenna.^10^ To be able to use the CMA for head imaging, these parameters therefore need to be optimized for operation at 596 MHz. To optimize parameters *d*, ℓ, and *L*, the antenna was modeled after a commercially available coaxial cable (Huber Suhner RG223u; characteristic impedance: 50 Ω). The coaxial cable's core and shield were modeled as a perfect electrical conductor. The outer diameters of the core, dielectric, shield, and jacket were set to 0.89, 2.95, 3.85, and 5.3 mm, respectively. The relative permittivity of the dielectric and the jacket were set to ε_r_ = 2.3 and ε_r_ = 4.0 respectively, to mimic the properties of a real coaxial cable. Both for the dielectric and the jacket, a conductivity of 0 S/m was used. The source was placed between the shield and the core of the coaxial cable. The combinations that were tested are *d* = 3, 4, and 5 cm, and ℓ = 16, 18, 20 cm. By using network cosimulation, the performance of the antenna can then be evaluated for a range of values for *L*, without the need to simulate each separate value.^13^ The simulation setup is shown in Figure 1D.

The CMAs were voxelized at a resolution of 2 mm in the z‐direction and 0.15 mm in the x‐y direction, ensuring sufficiently accurate voxelization of the circular structure of the antenna. A total of 2.830–3.054 Mcells (1 Mcell = 1.0 × 10^6^ cells) was needed, depending on antenna length ℓ.

#### 2.1.2 Fractionated dipole antenna

The fractionated dipole antenna was modeled by two perfect electrical conductor strips with zero thickness, variable length ℓ, and 10‐mm width, separated by a 2‐mm gap. Both sides of the antenna were segmented into three parts separated by 12‐mm gaps. In these gaps, meandered structures of 8 cm were added to mimic the designs commonly used at 7 T.^5^ The antenna length ℓ was varied between 20 and 30 cm with 2.5‐cm increments. The antennas were matched to the 50Ω input cable using a different matching network for each antenna.

The fractionated dipoles were voxelized at an isotropic resolution of 1 mm^3^, resulting in a total of 3.267–4.361 Mcells depending on ℓ.

### 2.2 Eight‐channel head simulations human model

Eight‐channel head simulations were performed on the head and shoulders of the realistic model Duke.^11^ From these simulations, the $B_{1}^{+}$ and SAR distributions for the two antenna arrays were obtained. The setups for these finite‐difference time‐domain simulations are shown in Figure 1E. As single‐channel results will show, the optimal configuration of the CMA is a length of 20 cm and a gap position of 4 cm. The most beneficial length of the fractionated dipole antenna is 25 cm. These antennas were placed in a circular array with a diameter of 26 cm and were driven with a phase difference of ¼π between each antenna. In both simulations, a radiofrequency shield with a radius of 310 mm and a length of 2 m is present. A bounding box with dimensions 315 × 450 × 320 mm^3^ ensures correct voxelization in the region of interest. The CMAs were voxelized at a resolution of 0.2 × 0.2 × 0.2 mm^3^. Because not all antennas could be aligned with the grid axes, not all fractionated dipole antennas had the same voxelization. The fractionated dipole antennas that were rotated over 45º had a voxelization of 0.35 × 0.35 × 1.5 mm^3^, whereas the antennas that were placed straight on the grid had a coarser voxelization of 1.5 × 1.5 × 1.5 mm^3^, to reduce the number of cells needed for the simulation. The chosen resolutions lead to a total of 39.289 and 21.263 Mcells for the CMA and fractionated dipole, respectively. A convergence criterion of −50 dB was used. Electric and magnetic fields and B_1_^+^ distributions were exported from *Sim4Life* into *MATLAB* (MathWorks, Natick, MA, USA). These fields were used to calculate 10 g–averaged Q‐matrices.^14^, ^15^, ^16^ These matrices were used to calculate the peak SAR levels for 100 000 random shim settings. A mask of the brain was created in which the average $B_{1}^{+}$ values were evaluated for the same random shim settings.

### 2.3 Coupling measurements

Eight CMAs and fractionated dipole antennas were constructed and placed onto a three‐dimensional (3D)–printed cylindrical holder with an inner diameter of 25 cm. At the center below the antennas, the cylinder had a thickness of 5 mm to obtain an outer diameter of 26 cm. Away from the antenna center, the cylinder was slightly thicker to create a flat surface to fully support the printed circuit boards (PCBs) of the fractionated dipole antennas. S‐parameter matrices of the arrays were acquired from 2 volunteers.

The CMA was built from RG402 50Ω Teflon coax cable. The cable traps were constructed from small PCBs and were tuned to 596 MHz using capacitors. The optimized dimensions serve as a guideline when building the antenna. The cable trap was placed over the coaxial cable at ℓ = 20 cm. At *d* = 4 cm, the shield was interrupted, and a hand‐wound inductor was used to connect the shield to the core of the antenna. By tuning this inductor, the antenna can then be matched to 50 Ω. Iterations with slight variations in ℓ and *d* were performed to arrive at the matched configuration. The antennas were placed onto the cylindric holder using zip ties.

The fractionated dipole was constructed from PCB (FR4). To match the antenna, two inductors with a value of 23 nH were soldered in series, and two capacitors (1 and 4.7 pF) were soldered in parallel to obtain a capacitance of 0.825 pF. The antennas were mounted to the cylinder using nylon bolts.

### 2.4 Flux probe measurements

To validate the simulation results, flux probe measurements were performed. A 32 × 28 × 10 cm^3^ phantom filled with water and 3 g/L salt was used for this experiment. The salinity of the water resulted in an approximate relative permittivity of 77 and a conductivity of 0.6 S/m. The antenna was placed under that phantom and connected to the primary port of a network analyzer (Keysight N9914A, Santa Rosa, CA, USA). The secondary port was connected to a flux probe (Tekbox Digital Solutions TBPS01–H20, Vietnam). Using a custom‐built automated positioning system, the probe was systematically moved through the phantom to create spatial maps of the S_21_ parameter, which reflects the efficiency distribution of the antenna.

In comparison to the intended use of the antennas (loading with a human head at a few centimeters distance), this phantom setup has some differences. The phantom material has a higher permittivity, while the phantom is also much larger and closer than the human head would be. As a result, the wavelength in the phantom and on the antenna is much shorter. The same measurement setup was therefore implemented in the simulation environment, to evaluate the agreement between the measured and simulated field distributions.

## 3 RESULTS

### 3.1 Single‐channel simulations

#### 3.1.1 Coax monopole

**FIGURE 3:**
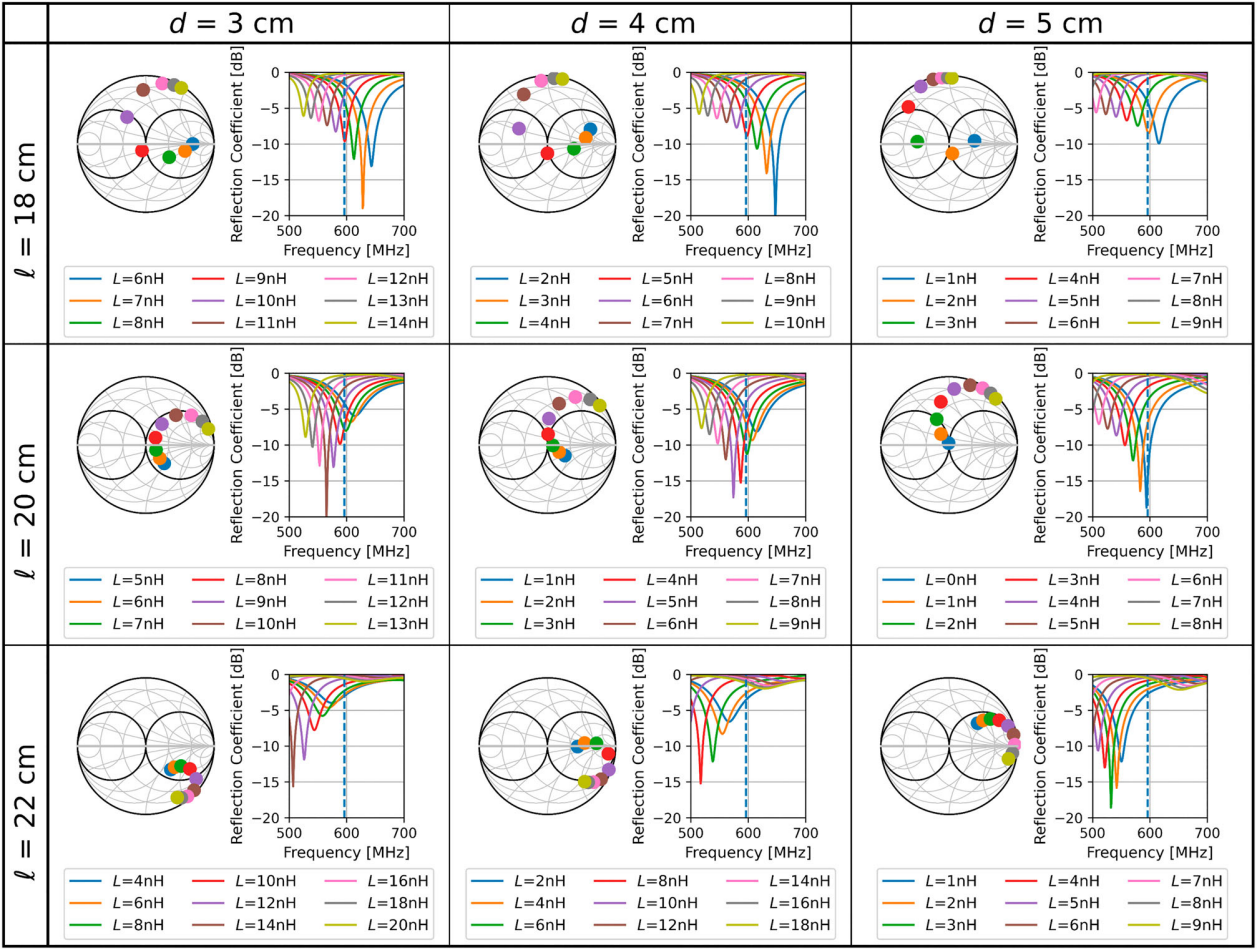
Smith charts and reflection plots for different combinations of length ℓ and gap position *d*. Each row represents a length, and each column represents a gap position. For each combination, the impedance and reflection have been investigated for a range of inductor values *L*. A gap position of 4 cm, a length of 20 cm, and an inductance of 3 nH deliver matching to 50 Ω.

Figure 3 shows Smith Charts and reflection plots for the investigated combinations of *d* (3 cm, 4 cm, 5 cm), ℓ (18 cm, 20 cm, 22 cm), and *L*. The Smith charts show that matching is not achievable for every combination. A combination of ℓ = 20 cm and *d* = 4 cm ensures the possibility of matching with an inductance of 3 nH, while maintaining the possibility of tweaking the value slightly to a higher or lower value.

#### 3.1.2 Fractionated dipole

**FIGURE 4:**
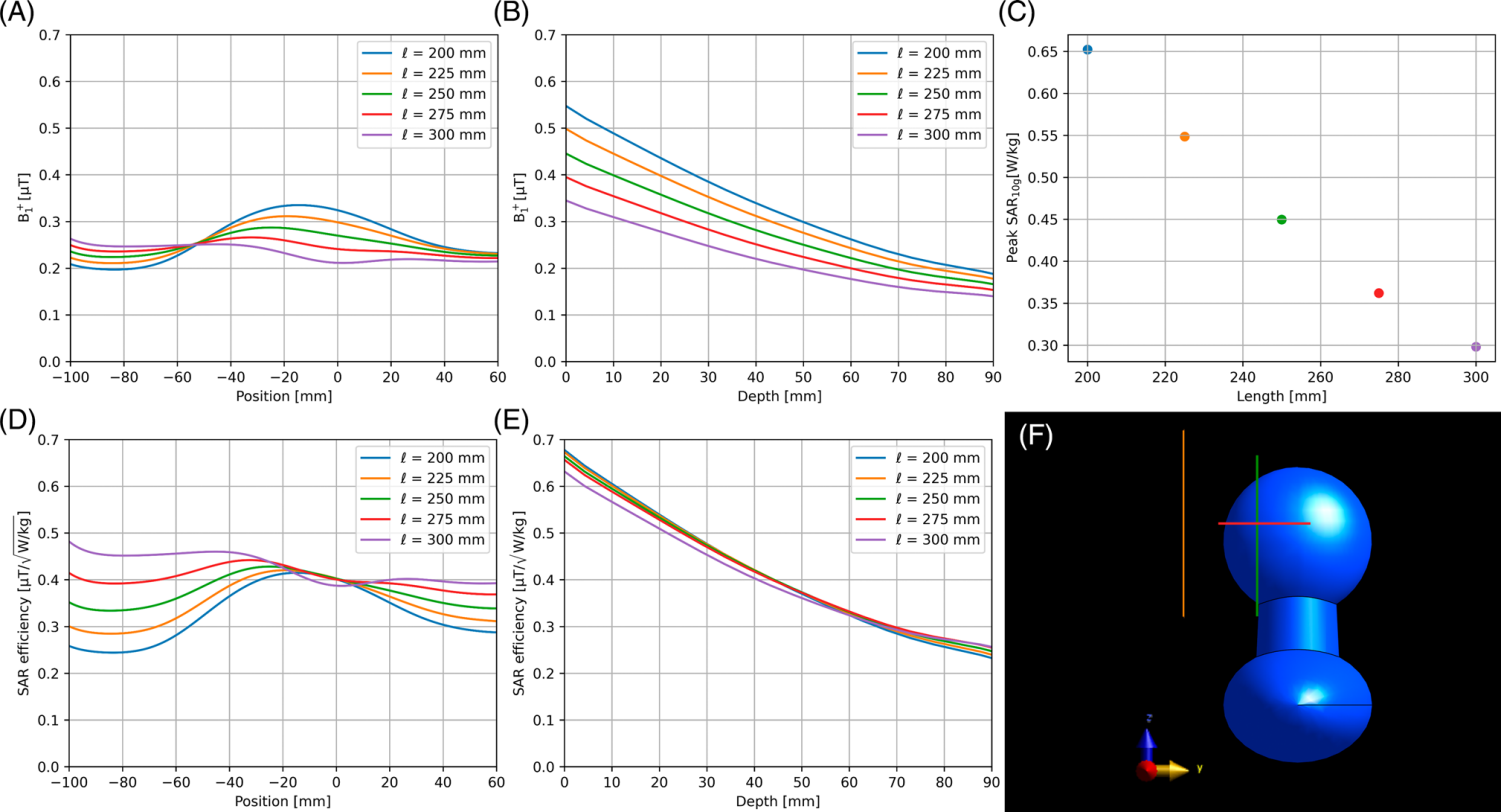
(A) $B_{1}^{+}$ profile in the longitudinal direction at 5‐cm depth for the fractionated dipole with different lengths, taken directly below the antenna. (B) In‐depth $B_{1}^{+}$ profile, taken directly below the antenna. (C) Specific absorption rate (SAR) levels for the fractionated dipole with different lengths. (D) SAR efficiency profile in the longitudinal direction at 5‐cm depth. (E) In‐depth SAR efficiency profile. (F) Simulation geometry in the *y*‐*z* plane. The green line indicates the position of the longitudinal profile in (A); the red line indicates the position of the depth profile in (B); and the orange line indicates the position of the antenna. The $B_{1}^{+}$ profiles and SAR levels were all normalized to 1 W of conducted power.

Figure 4A,B shows the longitudinal $B_{1}^{+}$ profiles at 5‐cm depth, and the in‐depth $B_{1}^{+}$ profiles, directly below the antenna, for the fractionated dipoles with different lengths ℓ (20, 22.5, 25, 27.5, and 30 cm). The locations of these profiles are indicated by the green and red lines in Figure 4F, respectively. The profiles show that the $B_{1}^{+}$ efficiency is highest for the shortest antenna, and the $B_{1}^{+}$ efficiency decreases when the antenna length is increased. Increasing the length to 25 cm results in a field distribution that is more homogenous than that of the shorter antennas. Increasing the length even further results in a dip in the center of the $B_{1}^{+}$ distribution, because of a second maximum appearing in the current distribution over the antenna: The antenna becomes too long with respect to its wavelength. The SAR levels decrease as the antenna length increases, as shown in Figure 4C. The resulting longitudinal and in‐depth SAR efficiency profiles are shown in Figure 4D,E, respectively. The SAR efficiency of the longest antenna is highest and decreases when the antenna length is decreased. The choice for the antenna length is thus a trade‐off among SAR efficiency, field homogeneity, and $B_{1}^{+}$ efficiency.

#### 3.1.3 Single‐channel comparison

**FIGURE 5:**
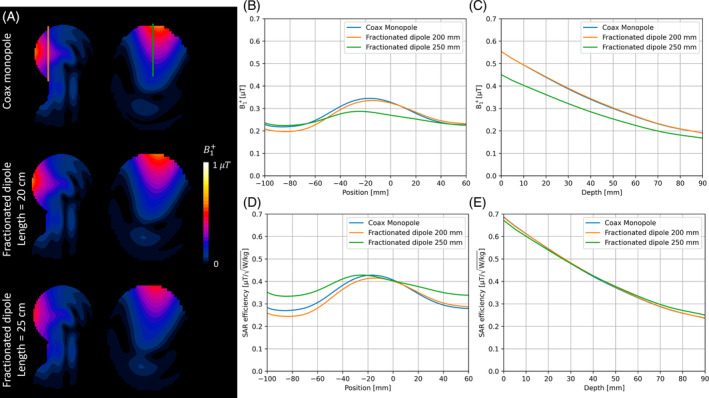
(A) Simulated $B_{1}^{+}$ maps for the coax monopole (*top*) and fractionated dipole with length 20 cm (*middle*) and fractionated dipole with length 25 cm (*bottom*). (B) Longitudinal $B_{1}^{+}$ profiles at 5‐cm depth in the phantom (location indicated by orange line in [A]). (C) In‐depth $B_{1}^{+}$ profiles (location indicated by the green line in [A]). (D) Longitudinal specific absorption rate (SAR) efficiency at 5‐cm depth. (E) In‐depth SAR efficiency. All $B_{1}^{+}$ and SAR profiles were normalized to 1 W of conducted power.

The design optimizations for the CMA and the fractionated dipole resulted in optimal lengths of ℓ = 20 cm for the CMA and ℓ = 25 cm for the fractionated dipole. This may be a potential source of bias. Particularly for the fractionated dipole, the length is a trade‐off, and depending on priorities, other lengths may be considered optimal. Therefore, the fractionated dipole will be evaluated for its optimal length of 25 cm and for a length of 20 cm, which is equal to the optimal CMA. The simulated $B_{1}^{+}$ maps for the CMA and fractionated dipole with ℓ = 20 cm and ℓ = 25 cm are shown in Figure 5A. The orange and green lines indicate the position of the profiles shown in Figure 5B,C, respectively. These profiles show that the $B_{1}^{+}$ efficiency of the CMA and the fractionated dipole with ℓ = 20 cm is comparable in both directions. The fractionated dipole with ℓ = 25 cm shows a more homogeneous field distribution, at the cost of a lower $B_{1}^{+}$ efficiency. SAR levels were 0.65, 0.65, and 0.45 W/kg for the CMA, fractionated dipole with ℓ = 20 cm, and fractionated dipole with ℓ = 25 cm, respectively. The resulting SAR efficiency profiles are found in Figure 5D,E. Although the maximum SAR efficiency of all three antennas is similar, the fractionated dipole with ℓ = 25 cm has a larger field of view than the other two antennas.

### 3.2 Eight‐channel simulation Duke

**FIGURE 6:**
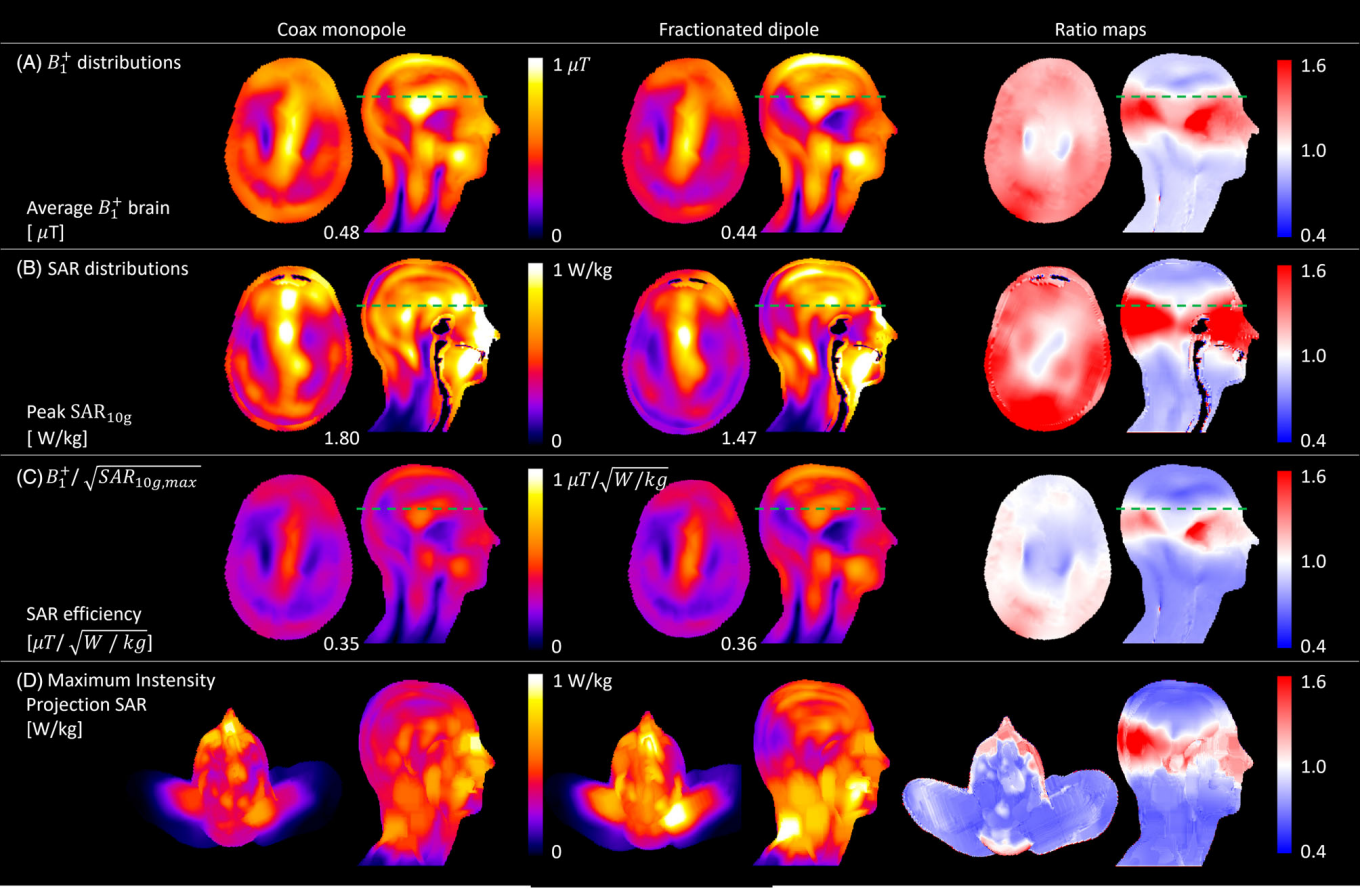
Coil array simulation results for coax monopole (*left*) and fractionated dipole (*middle*) and ratio maps of the CMA over the fractionated dipole (*right*). (A) $B_{1}^{+}$ distributions for 8 × 1 W conducted power. (B) Specific absorption rate (SAR) distributions for 8 × 1 W conducted power. (C) $B_{1}^{+}/\sqrt{\mathrm{SA}R_{10g,\max}}$ distributions. (D) Maximum intensity projection of the SAR levels. The location of the transversal slice is indicated by the green dotted line in the transversal plane.

For eight‐channel arrays loaded with the human model Duke, Figure 6A–D shows the simulated $B_{1}^{+}$ distributions, SAR distributions, and SAR efficiencies, and maximum intensity projection of the SAR levels, respectively, for the CMA (left) and fractionated dipole (middle) in the sagittal plane and transversal plane. The green dotted line indicates the location of the transversal slice. The right column shows the ratio of the CMA over the fractionated dipole antenna. The $B_{1}^{+}$ distributions and SAR distributions were normalized to 8 × 1 W conducted power. A mask was created using gray matter, white matter, cerebrospinal fluid, cerebellum, and corpus callosum. The average $B_{1}^{+}$ values in this mask were 0.48 and 0.44 μT for the CMA and fractionated dipole, respectively. In the same order, the 10 g–averaged peak SAR levels were found to be 1.80 and 1.47 W/kg, located in the eyes and the neck. This results in $B_{1}^{+}/\sqrt{\mathrm{SA}R_{10g,\max}}$ ratios of 0.35 and 0.36 $\mathrm{\mu T}/\sqrt{W/\mathrm{kg}}$. The ratio maps show that the $B_{1}^{+}$ levels, SAR levels, and SAR efficiency of the CMA are higher than those of the fractionated dipole in the middle of head, whereas the levels of the fractionated dipole are higher at the top of the head and the lower portion of the head.

**FIGURE 7:**
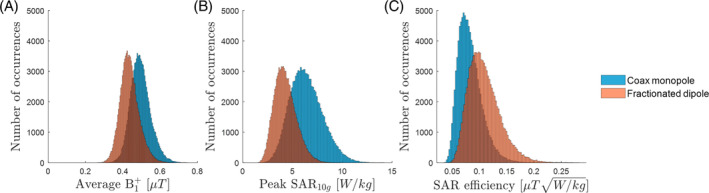
For 100 000 random phase settings for the coax monopole and fractionated dipole. (A) Average $B_{1}^{+}$ in the brain. (B) Peak specific absorption rate (SAR_10g_) levels. (C) SAR efficiency.

Figure 7A shows histograms of the average $B_{1}^{+}$ in the mask, for 100 000 random shims. The amplitude was fixed and normalized to 8 × 1 W input power and the phases were randomized between 0 and 2 π. The CMA reaches higher $B_{1}^{+}$ levels but has a broader spread. Figure 7B,C shows the peak SAR levels and the SAR efficiency for the same shim settings. The CMA shows higher SAR levels than those of the fractionated dipole, resulting in somewhat lower SAR efficiencies.

**FIGURE 8:**
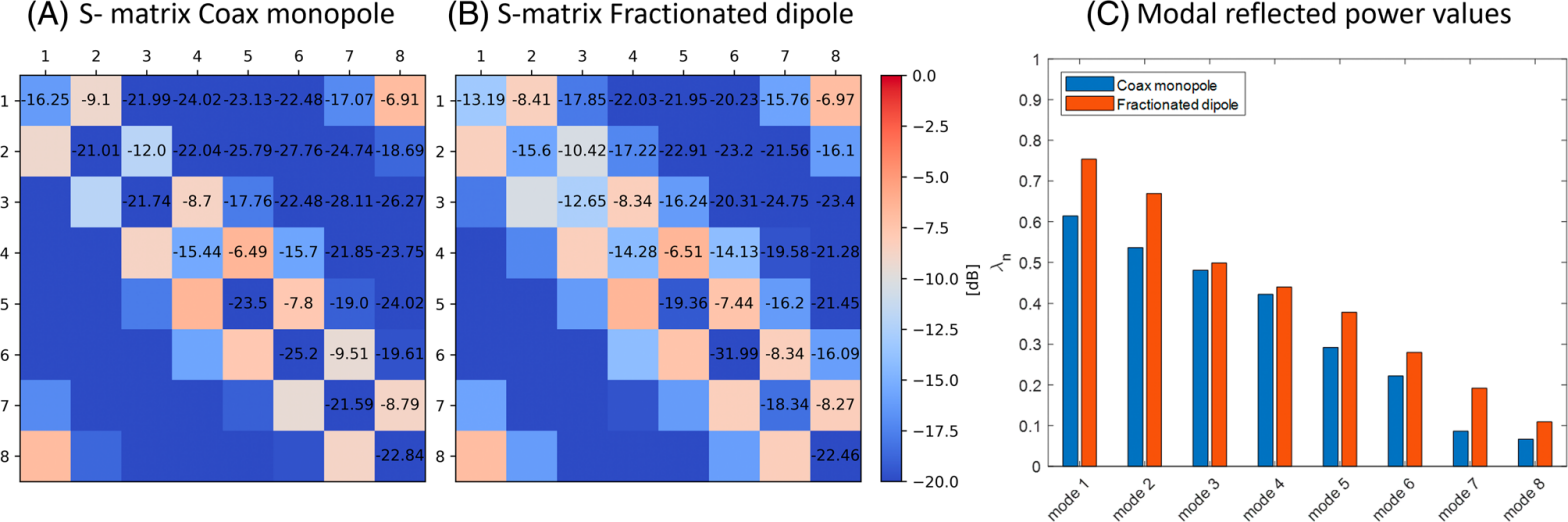
(A,B) Simulated scattering matrices for the coax monopole antenna (A) and fractionated dipole antenna arrays (B). (C) Modal reflected power values of the simulated scattering matrices.

The simulated S‐matrices for the two arrays are shown in Figure 8. The best S_11_ values were −25.2 dB for the CMA and −32.0 dB for fractionated dipole antenna. The worst S_11_ values were −15.4 dB and −12.7 dB for the CMA and fractionate dipole, respectively. The worst S_21_ values were −6.49 and −6.51 dB for the CMA and fractionated dipole, respectively, and the best values were, in the same order, −12.0 and −10.42 dB. For the simulated S‐matrices, eigenmode analysis was performed, as shown by Kazemivalipour et al.^17^ The resulting modal‐reflected power values are visualized in Figure 8C. The coax monopole shows lower modal‐reflected power values than the fractionated dipole, resulting in lower maximum reflections.

### 3.3 Coupling measurements

**FIGURE 9:**
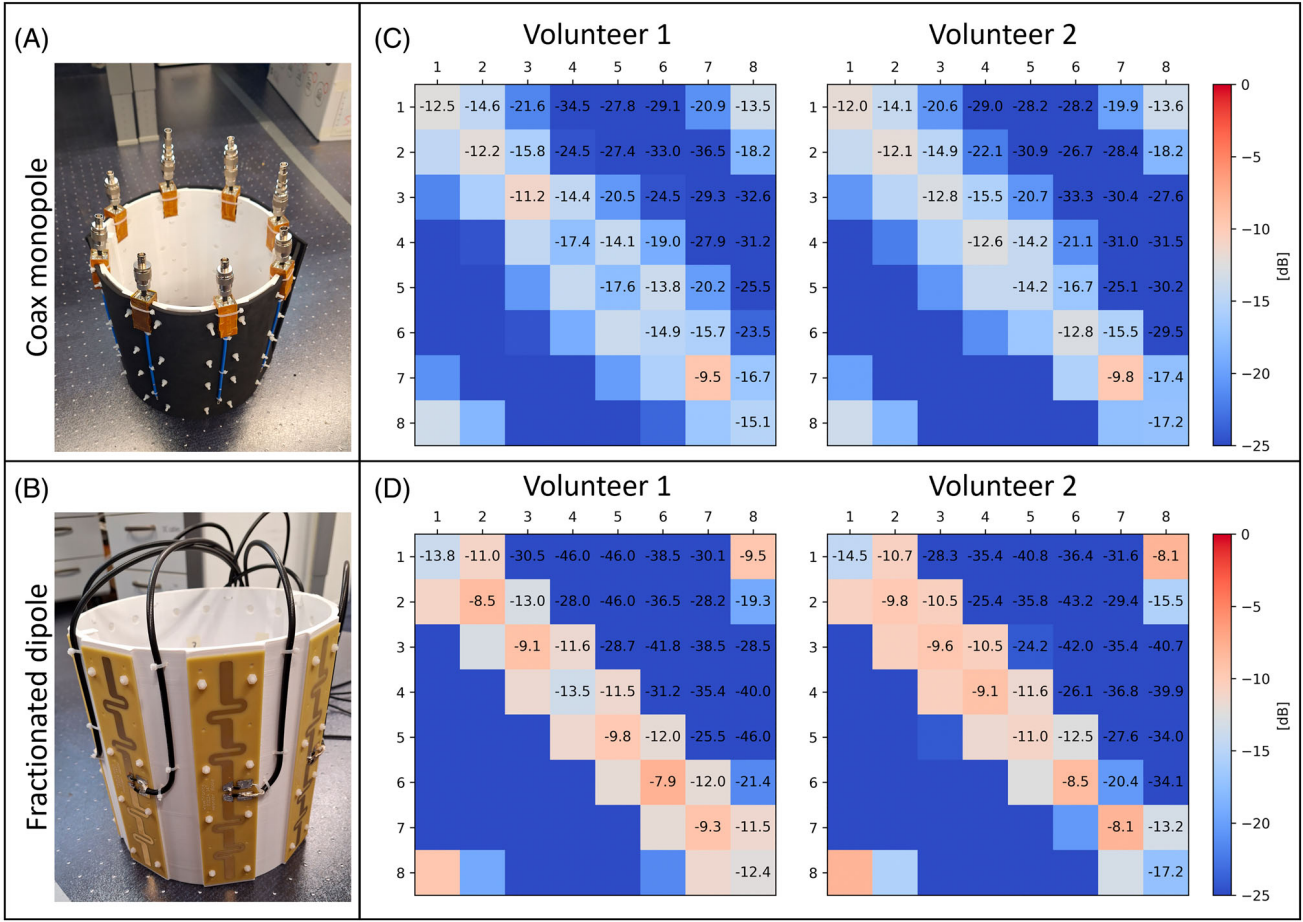
(A) Realization of an eight‐channel head array of coax monopole antennas. (B) Realization of an eight‐channel head array of fractionated dipole antennas. (C) Measured S‐matrix of the eight‐channel coax monopole antenna array for 2 volunteers. (D) Measured S‐matrix of the eight‐channel fractionated dipole array for 2 volunteers. For all measurements, Antennas 1–4 are placed in front of the head, and Antennas 5–8 are placed at the backside of the head.

For the CMA and fractionated dipole, eight elements were created and were mounted onto a 3D‐printed cylindrical holder with a diameter of 26 cm. The CMA showed considerable detuning after placing them onto the 3D‐printed tube. For this reason, a piece of ethylene‐vinyl acetate foam with a thickness of 10 mm was placed between the antenna and the tube to ensure a fixed distance between the CMA and the former. This effectively restored the matching of the antennas. The realized eight‐channel arrays are shown in Figure 9A. For both arrays, the S‐parameter matrices were obtained for 2 volunteers, as shown in Figure 9B. Antennas 1–4 were placed in front of the head, whereas Antennas 5–8 were placed at the backside of the head.

The worst S_11_ levels for the CMA were −9.5 and −9.8 dB for volunteers A and B, respectively, whereas the best levels were −17.6 and −17.2 dB. For the fractionated dipole, the worst S_11_ levels were −7.9 and −8.1 dB, and the best levels were −13.8 and −17.2 dB for Volunteers A and B, respectively.

The worst S_21_ levels for the CMA were −13.8 and −14.1 dB for Volunteers A and B, respectively, and the best levels (for nearest neighbors) were −16.7 and −17.4 dB. For the fractionated dipole, the worst S_21_ levels were −11.5 and −10.5 dB, and the best levels were −13.0 and −20.4 dB for Volunteers A and B, respectively.

### 3.4 Flux probe measurements

**FIGURE 10:**
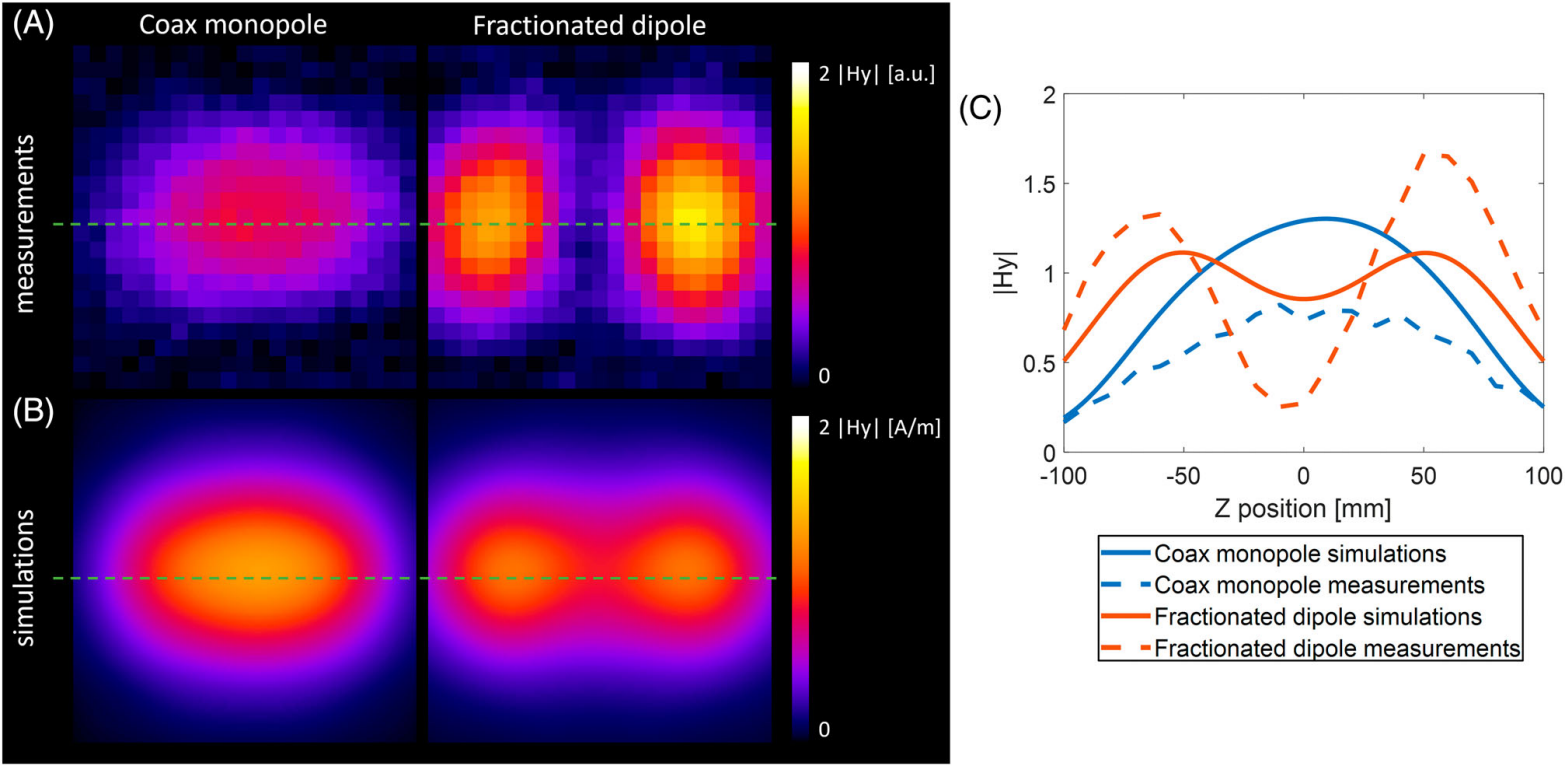
(A) Measured H‐field distributions for the coax monopole (*left*) and fractionated dipole antenna (*right*), in arbitrary units. (B) Simulated H‐field distribution of the coax monopole (*left*) and fractionated dipole (*right*) antenna, normalized to 1 W conducted power. (C) H‐field profiles, taken along the green lines in (A) and (B).

The H‐field distributions of the CMA and fractionated dipole are shown in Figure 10A for the measurements, and Figure 10B for the simulations. The CMA has one maximum, whereas the fractionated dipole antenna revealed two maxima in the H‐field distribution, due to the shorter wavelength and closer vicinity of the phantom. The simulated H‐fields show for both antennas the same patterns as for the measurements.

Figure 10C shows the profiles along the green lines of Figure 10A,B. The simulations suggest that the H‐field of the CMA is higher than that of the fractionated dipole in the center, and lower at the sides. Although the measurements show this as well, the maximum at the side lobes of the fractionated dipole is higher than the maximum in the center of the CMA. This suggests that the losses in the CMA are higher than those of the fractionated dipole.

## 4 DISCUSSION

In this research, the CMA and fractionated dipole antenna were optimized for head imaging at 14 T. The optimal configuration of the CMA was found to be a length of 20 cm, combined with a gap position of 4 cm. The optimal length of the fractionated dipole antenna was 25 cm.

In single‐channel simulations, the CMA had a higher $B_{1}^{+}$ efficiency than the fractionated dipole, with a shorter field of view. This is due to the length of the antenna. When comparing the CMA with the performance of a 20‐cm fractionated dipole, the CMA and fractionated dipole perform equally in terms of $B_{1}^{+}$ efficiency and SAR efficiency. However, increasing the length of the fractionated dipole to 25 cm resulted in a more homogeneous field distribution in the longitudinal direction but lower $B_{1}^{+}$ efficiency. Previous research at 7 T has suggested that the CMA has higher losses than the fractionated dipole.^10^ Similar to the simulations at 7 T,^10^ the simulations in this research were lossless. For the CMA, losses are expected in the insulators of the coax cable, and possibly in the cable trap as well. For the fractionated dipole, losses will occur in the FR4 substrate, which was excluded in the simulations. Expectations are that after including all losses in the simulations, the $B_{1}^{+}$ efficiency of the CMA will be like that of the 25‐cm fractionated dipole.

The results of the eight‐channel simulations indicate that the CMA and fractionated dipole perform similarly in terms of SAR efficiency when exited with ¼ π phase difference between the antennas. Histograms of 100 000 shims reveal that the CMA is more likely to perform less than the fractionated dipole antenna when driven with other phase settings. Histograms of 100 000 random shims reveal that the CMA, however, would perform less than the fractionated dipole antenna when driven with random phases in terms of SAR and SAR efficiency. The simulations appear to indicate higher $B_{1}^{+}$ levels for the CMA than for the fractionated dipole, but similar to the single‐channel simulations; this advantage is expected to diminish after including the losses.

For the calculation of the histograms, the data were not compressed into virtual observation points, as this has previously been shown to overestimate the peak SAR levels.^18^ Including a modern virtual‐observation‐point algorithm could drastically decrease computation time, while making sure that the SAR is not overestimated.

The simulated S‐parameter matrices show that both antenna arrays have acceptable reflection and coupling levels and are overall comparable. In the measured S‐parameter matrices, both the CMA and fractionated dipole antenna show increased reflection levels, compared with the simulation results, while showing decreased interelement coupling. The CMA shows both lower reflection as well as lower coupling levels with respect to the fractionated dipole antenna.

The main advantage of the CMA over the fractionated dipole antenna is its cable routing. Because the antenna is fed at one of the antenna endings, the routing of the feeding cables does not need to pass by any part of the antenna but will immediately leave the vicinity of the coil array at the top of the head. In contrast, the feeding cables of the fractionated dipole antenna have to be placed parallel to one of the antenna legs, which potentially results in cable‐coil coupling. This cable‐coil coupling is not well predictable. It is possible that a fractionated dipole array is built without any problems, if the cables are kept at distance from the antennas as much as possible. However, for denser coil arrays (e.g., 16 channels) and/or combination with insert gradients, the spatial constraints become more stringent, which would increase the likelihood of cable‐antenna interactions. In addition, recent studies at 10.5 T have shown considerable reduction in coil array efficiency due to cable‐coil coupling.^9^ These considerations make the CMA an attractive candidate for a head coil array design at 14 T.

Flux‐probe measurements were performed to validate the simulation results. The measured H‐fields showed similar patterns to the corresponding simulations, providing confidence in the simulation models of the antennas. The measurements also show that the losses in CMA are indeed higher than those in the fractionated dipole. A more quantitative assessment of these losses is expected when a 14T MR system is available.

The original intent of this study was to include also the folded‐end dipole into the comparison. This antenna has demonstrated a very good performance for 7T and 9.4T head arrays,^19^, ^20^ For our study, similar to the fractionated dipole antenna and the CMA, the design parameters of the folded dipole antenna were optimized. However, the optimization consistently showed that the most beneficial design has a folded segment of 0 cm (i.e., not folded at all). The folded‐end dipole is designed to profit from a more homogenous current profile when the antenna is shorter than λ/2.^19^, ^20^ Possibly, at 14 T, the antenna is not shorter than λ/2, and the additional folded segment does not provide an advantage. However, other explanations are possible as well, and we do not think that our simulation results should be used to draw definite conclusions. Nevertheless, for future reference, we have added our simulation results as Supporting Information.

## 5 CONCLUSION

In this work, the performance of the CMA has been evaluated for an eight‐channel transmit head array at 14 T and was compared with the fractionated dipole. Both the CMA and the fractionated dipole design parameters were optimized by single‐channel simulations. After this, the optimized designs were used to simulate an eight‐channel transmit array for head imaging using the human model Duke. The CMA showed slightly higher $B_{1}^{+}$ efficiency, but this advantage is expected to diminish if realistic losses would have been included. A prototype version of both arrays was built to evaluate reflection and coupling levels, which were generally good. Although the CMA is expected to perform with lower SAR efficiency than the fractionated dipole antenna, the CMA will provide easier cable routing, as its feeding point is at the proximal side of the antenna instead of at the center.

## CONFLICT OF INTEREST

Ingmar Voogt is cofounder of WaveTronica B.V. The other authors declare no conflicts of interest.

## Supporting information


**Figure S1.** (A) Simulation geometry in y‐z plane. The green line indicates the position of the longitudinal profiles, used in Figure S1C. (B) Simulation geometry in x‐z plane. The red dot indicates the position of the in‐depth profiles, used in Figure S1A. (C) Simulation geometry in the x‐y plane. The red line indicates the position of the in‐depth profiles, and the green dot indicates the position of the longitudinal profiles, used in Figure S1A,B, respectively.
**Figure S2.** A folded dipole with varying heights (H) and folds (F), with base length 18 cm (*right*), 20 cm (*middle*), and 22 cm. (A) $B_{1}^{+}$ profiles in the longitudinal direction at 5‐cm depth, normalized to 1W conducted power. The location is indicated by the green lines in Figure S1. (B) Specific absorption rate (SAR) efficiency in the longitudinal direction at 5‐cm depth. (C) In‐depth $B_{1}^{+}$ profiles, normalized to 1 W of conducted power. The location is indicated by the red lines in Figure S1. (D) In‐depth SAR efficiency profiles. (E) Peak SAR levels, normalized to 1 W of conducted power.
