# Supplementary material for: An evaluation of the coax monopole antenna as a transmit array element for head imaging at 14 T
Source: Magn Reson Med. 2025 Feb 18;93(6):2667–79. doi: 10.1002/mrm.30464 (PMC11971495; doi:10.1002/mrm.30464)
Supplement: Supplementary file 1 — Figure S1. (A) Simulation geometry in y‐z plane. The green line indicates the position of the longitudinal profiles, used in Figure S1C. (B) Simulation geometry in x‐z plane. The red dot indicates the position of the in‐depth profiles, used in Figure S1A. (C) Simulation geometry in the x‐y plane. The red line indicates the position of the in‐depth profiles, and the green dot indicates the position of the longitudinal profiles, used in Figure S1A,B, respectively. Figure S2. A folded dipole with varying heights (H) and folds (F), with base length 18 cm (right), 20 cm (middle), and 22 cm. (A) B1+ profiles in the longitudinal direction at 5‐cm depth, normalized to 1W conducted power. The location is indicated by the green lines in Figure S1. (B) Specific absorption rate (SAR) efficiency in the longitudinal direction at 5‐cm depth. (C) In‐depth B1+ profiles, normalized to 1 W of conducted power. The location is indicated by the red lines in Figure S1. (D) In‐depth SAR efficiency profiles. (E) Peak SAR levels, normalized to 1 W of conducted power. [file MRM-93-2667-s001.pdf]

Supporting information

Optimization folded-end dipole antenna

The folded-end dipole antenna has three dimensions that determine the performance of the antenna: the height (H), the fold (F), and the baselength of the antenna. These dimensions are indicated in Fig. S1a. To optimize these dimensions, multiple single-channel simulations were used. In these simulations, the baselength of the antenna was set to 18, 20, or 22 cm, and the height (H = 10, 20, 30, and 40 mm) and the size of the folded portion (F = 20, 40, 60, and 80 mm) were altered. For the antenna with baselength 200 mm, two extra fold sizes (F = 0 and 10 mm) were evaluated. The simulation setup is shown in Fig. S1, and is the same as the geometry used for the optimization of the CMA and fractionated dipole antenna. The conductor of the antenna was simulated as a PEC wire with thickness 1.5 mm. The folded-end dipole was voxelized at a resolution of 0.25 x 0.25 x 0.25 mm^3, and the phantom was voxelized at a maximum resolution of 5 x 5 x 5 mm^3.

Fig. S2a shows the resulting longitudinal B_1^+ profiles at 5 cm depth, directly below the antenna. The location of the profiles is indicated by the green lines in Fig S2. These results show that the B_1^+ magnitude becomes higher when the folded portion is smaller, and when the height of the antenna is decreased. The SAR decreases when the fold of the antenna becomes larger, and decreases when the height of the antenna is larger, as shown in Fig. S2e. The resulting SAR efficiency profiles in the longitudinal direction are shown in Fig. S2b. The highest SAR efficiencies are reached when folds and heights are as small as possible. Secondly, the profiles seem to indicate that a second mode appears on the antenna when the total conductor length becomes too large. Clear dips in the profiles are shown for the antennas with large folds and large heights.

In Fig. S2c, the in-depth profile, directly below the antenna are shown. The location is indicated by the red line in Fig. S1. The resulting SAR efficiencies are shown in Fig. S2d. Here, the same observations can be made as from the longitudinal profiles: The highest SAR efficiencies are reached when folds and heights are as small as possible. The SAR efficiencies are comparable for all configurations of the antenna.

These results seem to indicate that the optimal folded-end dipole antenna would be a dipole with a height of 0 mm and a fold of 0 mm, which is in essence a plain dipole. A possible explanation is given by the much shorter wavelength. The folded-end dipole antenna has been designed for situations where half the wavelength $\lambda/2$ is longer than the antenna. In those scenarios, the current distribution is peaked, rather than half a sinusoid. The folded segment of the antenna allows to restore the half-sinusoid current distribution resulting in a much more flat current distribution in the main (non-folded

segment). At 14T, the wavelength may have become so short that the folded segment of the antenna does no longer provide an advantage.

The folded-end dipole design could be advantageous for shorter antennas. However, these antennas would lead to a smaller field-of-view, which may render the antenna unsuitable for eight-channel head imaging. However, in a staggered 16-channel array, shorter folded-end dipole antennas might show some benefits. Also, this analysis is based on a single-element evaluation. For antennas in an array setup, particularly in combination with an RF shield, the optimal dimensions may be different.

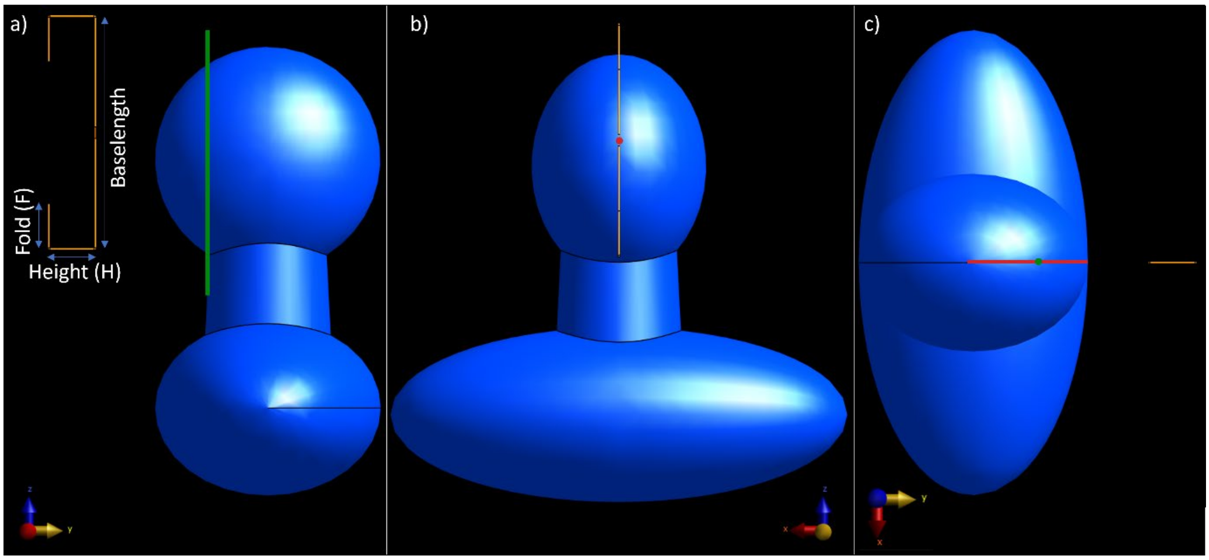

Fig. S1: a) Simulation geometry in y-z plane. The green line indicates the position of the longitudinal profiles, used in Fig. S2c. b) Simulation geometry in x-z plane. The red dot indicates the position of the in-depth profiles, used in Fig. S2a. c) Simulation geometry in the x-y plane. The red line indicates the position of the in-depth profiles, and the green dot indicates the position of the longitudinal profiles, used in Fig. S2a and b, respectively.

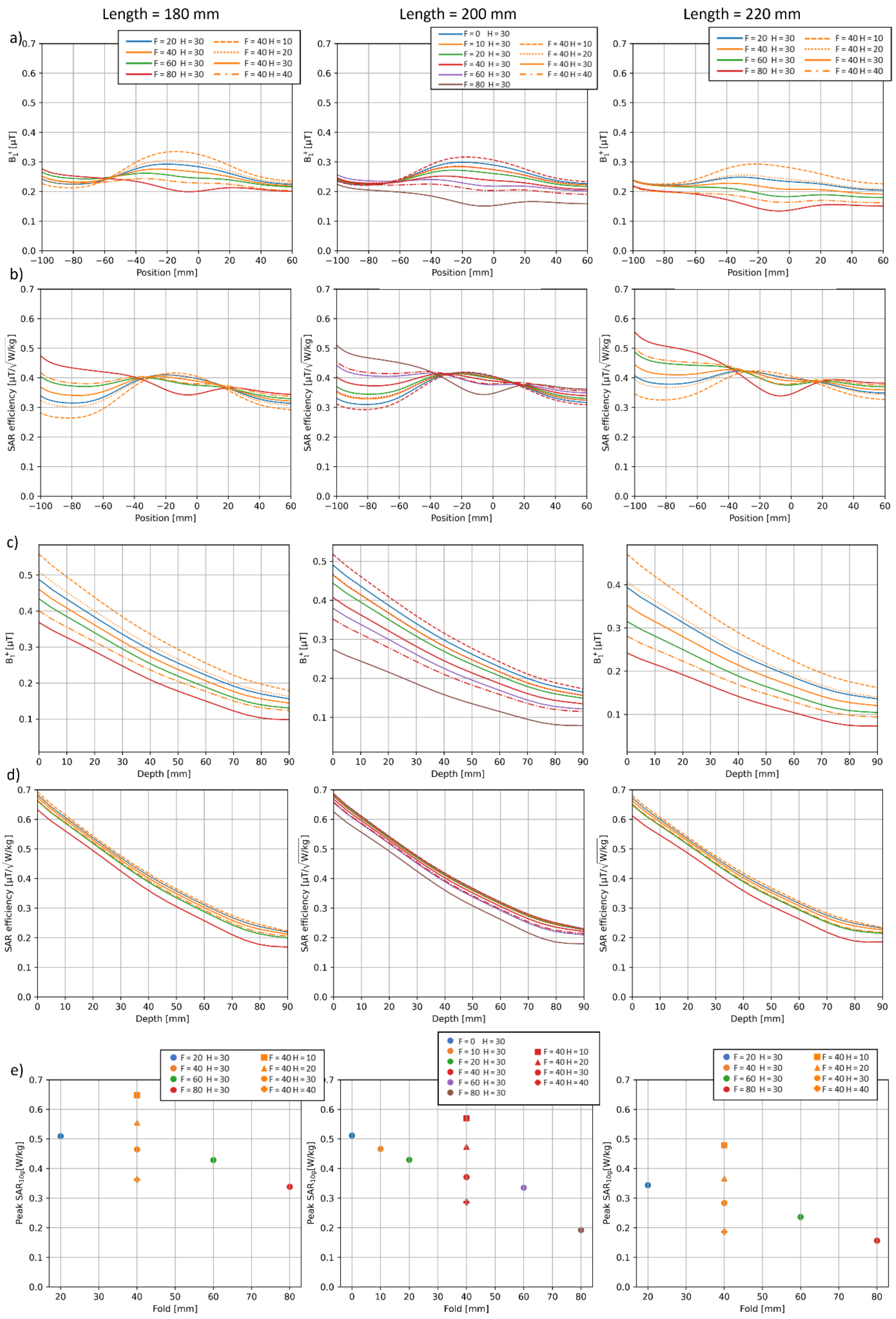

Length = 180 mm
Length = 200 mm
Length = 220 mm
a)
b)
c)
d)
e)
F = 0 H = 30
F = 10 H = 30
F = 20 H = 30
F = 40 H = 30
F = 60 H = 30
F = 80 H = 30
F = 40 H = 10
F = 40 H = 20
F = 40 H = 30
F = 40 H = 40
B_1^+ [µT]
SAR efficiency [µT/√W/kg]
Peak SAR_{10g}[W/kg]
Position [mm]
Depth [mm]
Fold [mm]

Fig. S2: For a folded dipole with varying heights (H) and folds (F), with base-length 18 cm (right), 20 cm (middle), and 22 cm; a) B_1^+ profiles in the longitudinal direction at 5 cm depth, normalized to 1 W conducted power. The location is indicated by the green lines in Fig. S1 b) SAR efficiency in the longitudinal direction at 5 cm depth c) In-depth B_1^+ profiles, normalized to 1 W conducted power. The location is indicated by the red lines in Fig. S1 d) In-depth SAR efficiency profiles e) Peak SAR levels, normalized to 1 W conducted power
